# Incidence, risk factors and impact of protocolised care on exposure keratopathy in critically ill adults: a two-phase prospective cohort study

**DOI:** 10.1186/s13054-017-1925-5

**Published:** 2018-01-16

**Authors:** Obaid Kousha, Zubaid Kousha, Jonathan Paddle

**Affiliations:** 1 Critical Care Unit, Royal Cornwall Hospital, Royal Cornwall Hospitals NHS Trust, Treliske, Truro, TR1 3LJ UK; 2 UCL Medical School, University College London, Gower Street, London, WC1E 6BT UK

**Keywords:** Clinical protocols, Corneal injuries, Critical care

## Abstract

**Background:**

Exposure keratopathy (EK) has a high incidence in critically ill patients. We aimed to determine the rate of EK in patients admitted to our intensive care unit (ICU), identify risk factors for developing EK and ascertain the effectiveness of a protocol to prevent EK.

**Methods:**

We undertook a two-phase prospective cohort single-centre study in a general adult ICU. The first phase of the study was observational. In the second phase of the study an eye care protocol was introduced. Daily ophthalmic assessment was carried out using a portable slit lamp. We also recorded Acute Physiology and Chronic Health Evaluation II score, daily Sequential Organ Failure Assessment score, mechanical ventilation, Richmond Agitation-Sedation Scale, and level of eye care. Student’s *t* test and *χ*^*2*^ statistics were used for simple analysis of continuous data and categorical data, respectively. Binary logistic regression was used to analyse the relationship between EK (yes/no), as the dependent variable, and multiple independent variables, calculating unadjusted and adjusted odds ratios.

**Results:**

We studied 371 patients. In the first phase, the overall rate of EK was 21% but the rate in mechanically ventilated patients was 56%; *χ*^*2*^ (1, *N* = 257) = 80.8, *p* < 0.001. Adjusted odds ratios (AOR) for development of EK were 28.6 (8.19–43.37), 13.0 (3.16–54.38) and 1.2 (1.03–1.33) with incomplete eye closure, mechanical ventilation, and higher SOFA score, respectively. Following the introduction of the protocol in the second phase, the overall rate of EK reduced to 2.6% (three cases); *χ*^*2*^ (1, *N* = 371) = 18.6, *p* < 0.001. Compliance with the protocol was 97%.

**Conclusions:**

EK is common in critically ill patients, and is associated with mechanical ventilation and incomplete eye closure. A simple protocol substantially reduces the incidence of EK and is easily achieved in clinical practice.

## Background

**Fig. 1:**
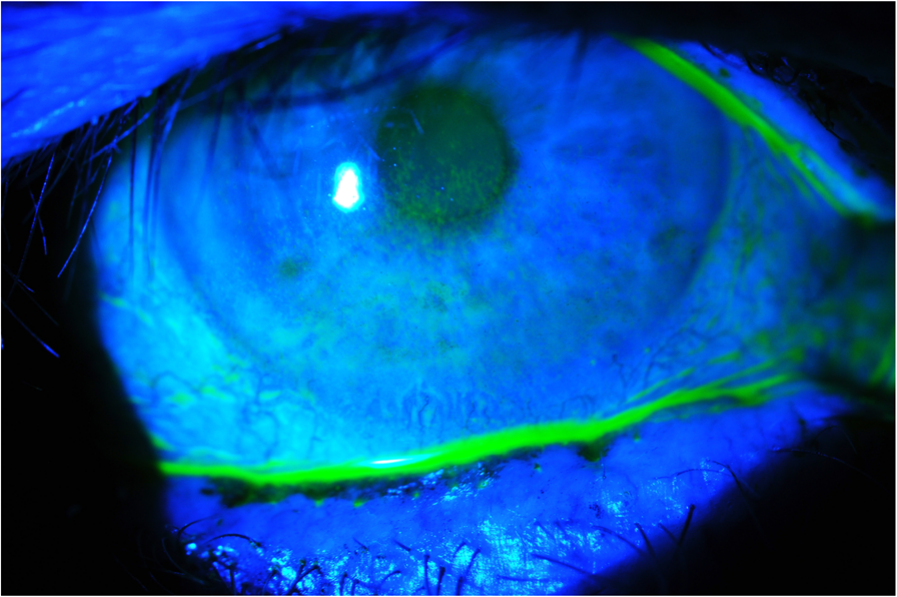
Exposure keratopathy due to lagophthalmos - fluorescein stain demonstrating extensive inferior corneal epithelial punctate erosion

Exposure keratopathy (EK) (Fig. 1) is a clinical syndrome characterised by incomplete eye closure and tear film defect leading to corneal damage of a spectrum of severity and extent [1]. A myriad of risk factors predisposes critically ill patients to EK, ranging from iatrogenic causes such as mechanical ventilation [2], sedation [3, 4] and muscle relaxants [5], to patient factors such as reduced consciousness [3], reduced tear production [6–8], reduced blink rate [6, 9], impaired corneal reflex [8, 10], incomplete eye closure [2, 11] and vascular permeability [10]. The EK rate in critically ill patients has been reported as low as 10% to as high as 55–60% [2]. Although usually EK resolves once the patient recovers, it is a distressing condition and can cause corneal scarring and permanent visual loss [12]. At worst, EK precipitates microbial keratitis leading to acute perforation, endophthalmitis and permanent visual impairment [13].

In view of this, efforts have been made to prevent EK [14]. Many strategies such as moisture chamber, lubrication and artificial tears have been developed [15–19]. Nevertheless, in an environment where the patient is suffering from acute life-threatening conditions, there is a lack of attention to issues such as EK, which may carry no immediate threat [20]. Therefore, a multidisciplinary focused approach is needed if EK is to be prevented, rather than simple prescriptions. Simply raising awareness of eye care through education of healthcare professionals working in an ICU reduces the rate of EK substantially without any changes to prescribed treatment [21]. Furthermore, formalised guidelines and protocols, when incorporated in patient care plans, can significantly reduce the rate of EK [22, 23].

Guidelines or protocols need to be simple to be effective. Significant adherence issues arise with detailed and complicated protocols [14]. The most pertinent risk factors need to be identified to develop a simplified eye assessment and management plan. However, to date, there is no widely accepted eye care protocol for prevention of EK in critically ill patients.

## Methods

### Aim

First, we aimed to determine the rate of EK in patients admitted to the ICU and to identify the risk factors for developing EK. Second, using the identified risk factors and experience from the first part of the study, we developed an eye care protocol. Third, we studied the effectiveness of this protocol to prevent EK.

### Study design

We undertook a two-phase prospective cohort single-centre study between November 2014 and August 2015 in a 19-bed general ICU in a large district general hospital (The Royal Cornwall Hospital NHS Trust, Cornwall, UK). The first phase of the study was primarily observational and was conducted from November 2014 to March 2015. However, in the course of the observational phase of the study when a patient was identified requiring further eye care, we intervened as needed. In the second phase of the study, from June 2015 to August 2015, we introduced an eye care protocol.

### Study population

All sequential patients admitted into the ICU were considered for inclusion into the study within 24 hours of admission in both phases of the study. Exclusion criteria were age <16 years, established pre-existing external eye disease, patient too agitated to tolerate the assessment, and patient’s or relative’s refusal to participate in the study. The patient remained as part of the study for the duration of their stay in the unit, unless palliated, which was treated as discharge from the unit.

### Data collection

**Fig. 2:**
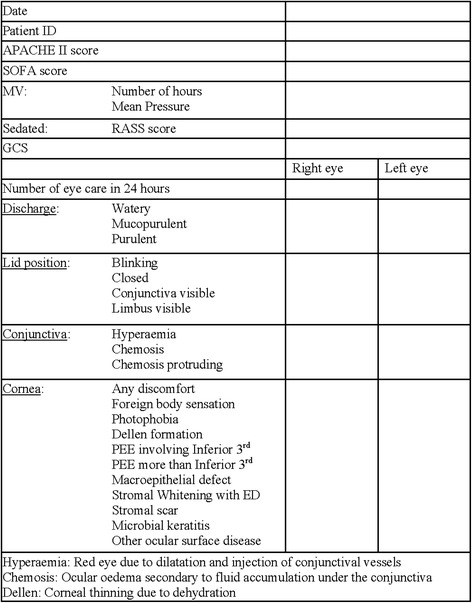
Data collection pro forma

All data were collected by one investigator (OK) trained in using a portable slit lamp using a pro forma (Fig. 2) adopted but modified from Mercieca et al. [3]. Data were collected every day from every patient throughout their stay on the ICU. Data collection involved accessing the patient’s electronic and paper record to exclude established external eye disease. The electronic patient record was used to calculate the patient’s Acute Physiology and Chronic Health Evaluation II (APACHE II) score for the admission and the daily Sequential Organ Failure Assessment (SOFA) score. Information related to mechanical ventilation, sedation including the Richmond Agitation-Sedation Scale (RASS), Glasgow Coma Score and eye care were collected at the bedside. Ophthalmic assessment was full examination of the external eye, eyelids including eyelid position and ocular surface using a portable slit lamp (Keeler PSL Classic). Ocular surface examination was carried out pre and post instillation of fluorescein dye (Minims Fluorescein sodium 2% w/v single-use preservative-free, manufactured by Bausch & Lomb UK Ltd). The pro forma was completed at the patient’s bedside.

### Eye care protocol

**Fig. 3:**
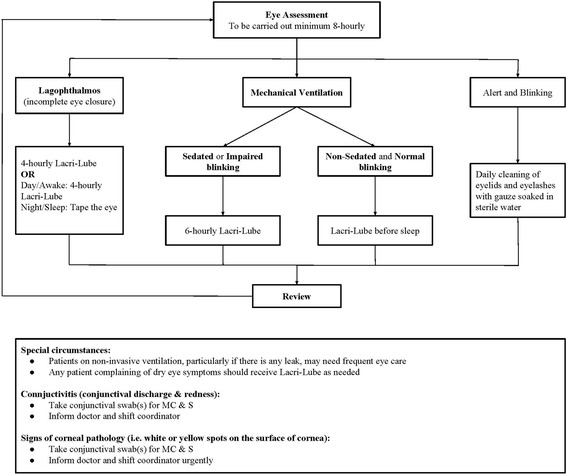
Eye care protocol

In the first phase of the study, before the development of an eye care protocol, eye care was left to the discretion of the bedside nurse until the patient developed EK. EK was defined as damage detected on the corneal surface using the fluorescein dye staining method, which was developed in the unit, except for microbial keratitis. All EK were managed upon detection. Punctate epithelial erosions and macro-epithelial defects were treated without referral to an ophthalmologist. Patients with corneal stromal whitening and scarring or non-resolving corneal signs were referred to an ophthalmologist. We developed the eye care protocol (Fig. 3) based on the risk factors identified and experience gained in the first phase of the study. The data from the first phase of the study were analysed, risk factors identified, level of treatment agreed upon and an eye care protocol produced, which was adopted by the unit after multidisciplinary team discussion. The eye care protocol was incorporated into the electronic record keeping and prescribing system.

### Statistical analysis

Statistical analysis was performed using IBM SPSS Statistics for Windows, Version 22. The tests were two-tailed with type I error set at α = 0.05. When analysing continuous data, two independent means were compared using Student’s *t* test. To analyse the relationships between binary categorical data, *χ*^*2*^ statistics were used. Binary logistic regression was used to analyse the relationship between EK (yes/no), as the dependent variable, and multiple independent variables, calculating unadjusted and adjusted odds ratios (ORs). The adjusted ORs were calculated to determine the effect of each risk factor independently when all else was equal. For the adjusted model, collinearity of the independent variables was tested by testing for correlation and if two variables were found to be strongly correlated, one was removed from the model to avoid model instability. We used Pearson correlation for parametric data, Spearman’s rank correlation for non-parametric non-binary data and the phi-coefficient for non-parametric binary data. To assess how much variation in the dependent variable is explained by the model, Nagelkerke *R*^*2*^ was used.

## Results

**Fig. 4:**
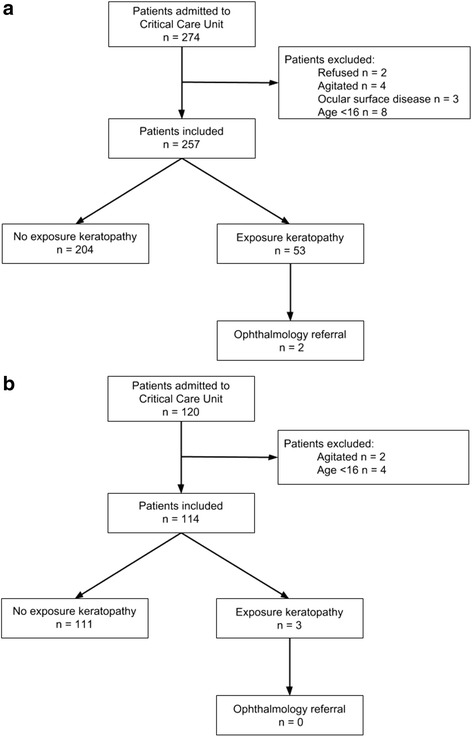
Flow diagram for the first (**a**) and second (**b**) phase of the study

There were 371 patients included in this study, with 257 included in the first phase and 114 included in the second phase (Fig. 4). Patient characteristics in the control group and the intervention group were well-matched (Table 1).

**Table 1:** Characteristics of patients in the control and intervention groups

	Control	Intervention	*P* value
Patients (*n*)	257	114	
Eye assessment (*n*)	2712	980	
Male (*n*)	123 (48%)	62 (54%)	0.246
Age (years)	62 (17)	63 (17)	0.759
APACHE II score	16 (7)	17 (7)	0.547
Median SOFA score	3 (1.5–5.0 (0–15))	3 (1–4.75 (0–18.5))	0.792
Mechanically ventilated (*n*)	80 (31%)	33 (29%)	0.674
Average P_mean_	3.1 (4.9)	2.9 (4.6)	0.396
Chemosis (*n*)	37 (14%)	13 (12%)	0.216
Sedated (*n*)	83 (32%)	30 (26%)	0.248
Incomplete eye closure (*n*)	25 (10%)	11 (9.6%)	0.981
EK (*n*)	*53 (21%)*	*3 (2.6%)*	*<0.001*
• PEE involving Inferior 3^rd^ • PEE more than Inferior 3^rd^ • Macro-epithelial defect • Stromal whitening with ED • Stromal scar	30 (11.7%) 12 (4.7%) 9 (3.5%) 1 (0.4%) 1 (0.4%)	3 (2.6%)	
Average resolution of EK in days	*3.73 (4.11)*	*1.25 (0.5)*	*<0.001*
Referred to ophthalmology (*n*)	2 (1%)	0	
Compliance with protocol (*n*)	N/A	111 (97%)	

Values are mean (SD), median (IQR (range)) or number (proportion) unless stated otherwise

*APACHE II* Acute Physiology and Chronic Health Evaluation II, *SOFA* Sequential Organ Failure Assessment, *P*_*mean*_ mean ventilation pressure, *EK* exposure keratopathy, *PEE* punctuate epithelial erosion, *ED* epithelial defect, *N/A* Not applicable

### First phase

The first phase of the study was conducted to ascertain the rate of and the risk factors for development of EK. The overall rate of EK was 21%. Among mechanically ventilated patients the rate was 54.3% compared to 5.1% in patients receiving non-invasive ventilation or no ventilatory support, *χ*^*2*^ (1, *N* = 257) = 80.8, *p* < 0.001: 69% of EK developed in the first 48 hours of admission.

To further elucidate the contribution of age, APACHE II score, SOFA score, ventilation status, mean ventilation pressure (P_mean_), chemosis, sedation and incomplete eye closure to the development of EK, unadjusted and adjusted ORs were calculated using a binary logistic regression model (Table 2). Although unadjusted ORs identify multiple risk factors, this is narrowed down when ORs are adjusted (Table 2). Hence, when all else is equal, incomplete eye closure, mechanical ventilation or one point increase in median SOFA score each independently increase the odds of developing EK by a factor of 28.6 (8.19–43.37), 13.0 (3.16–54.38) and 1.2 (1.03–1.33) respectively. Sedation was omitted from the adjusted ORs due to severe collinearity with mechanical ventilation but other factors had no independent statistically significant effect on EK (Table 2).

**Table 2:** Study phase I: odds ratios (OR) of developing exposure keratopathy in control group for various factors

Risk factors	Unadjusted OR (95% CI)	Adjusted OR (95% CI)
Age^a^	1.00 (1.00–1.03)	1.02 (1.00–1.04)
Average APACHE II score^b^	1.13 (1.09–1.18)***	1.04 (0.98–1.10)
Median SOFA score^c^	1.41 (1.31 –1.58)***	1.22 (1.03–1.33)*
Mechanical ventilation	22.8 (12.0–46.5)***	13.0 (3.16–54.3)***
Average P_mean_^d^	1.29 (1.22–1.37)***	0.946 (0.826–1.08)
Chemosis	3.30 (1.41–6.23)***	1.30 (0.51–5.53)
Sedation	24.6 (12.7–51.6)***	Omitted^e^
Incomplete eye closure	77.4 (25.8–347)***	28.6 (8.19–43.4)***

Binary logistic regression model for adjusted OR had Nagelkerke *R*^*2*^ = 0.58

*APACHE II* Acute Physiology and Chronic Health Evaluation II, *SOFA* Sequential Organ Failure Assessment, *P*_*mean*_ mean ventilation pressure

^a^Increase in age of 1 year

^b^Increase in APACHE II score of 1 point

^c^Increase in median SOFA score of 1 point

^d^Increase in average P_mean_ of 1 cmH_2_O

^e^Due to severe collinearity between mechanical ventilation and sedation (Phi coefficient (*r*_*φ*_) = 0.95 *p* value <0.001), sedation was omitted from the analysis to avoid instability of the model

*P value <0.05; ***p value <0.001

The majority of the cases of EK were punctate epithelial erosions, a mild form of EK (Table 1). All but one of the patients who developed EK were managed successfully without long-term sequelae. Of the 53 patients with EK, 51 were successfully managed in the unit and 2 required specialist ophthalmology input. In one case permanent scar to the cornea was sustained but no patients developed microbial keratitis.

### Second phase

Following institution of the protocol in the second phase of the study, the rate of EK was significantly reduced. Overall, compared to the 21% rate of EK in the first-phase cohort, only 2.6% of patients (n = 3) developed EK, *χ*^*2*^ (1, *N* = 371) = 18.6, *p* < 0.001. On average, the time of resolution of EK was three times longer in the first phase of the study as compared to the second phase (Table 1).

The adherence rate to the eye care protocol was 97%. There were three cases of EK in the second-phase cohort (Table 1). In two of these cases, both in mechanically ventilated patients, the eye care protocol was not started. In the third case, a mechanically ventilated patient who subsequently developed incomplete eye closure during his stay in the ICU, the eye care protocol was started initially at the correct step but eye care was not escalated as per protocol when the patient developed incomplete eye closure. All patients with EK were successfully managed in the unit and no speciality referral was required in the second phase of the study.

## Discussion

In the largest study of its kind to date, we have shown that the introduction of protocolised eye care in critically ill patients, developed in conjunction with the multidisciplinary team, substantially reduced the incidence and duration of EK. It was the first study to date to assess the development of EK in patients admitted to the ICU pre and post implementation of protocolised eye care. Early detection, treatment and daily follow up of EK meant that the majority of patients developed only a mild form of EK, with quick resolution. However, if left untreated, mild EK is highly likely to progress to a more severe form.

In the first-phase, the overall rate of EK, its timing of development and its rate in patients with mechanically ventilated lungs were similar to the findings of others [2, 24, 25]. Studies reporting lower prevalence are either retrospective in nature [8] or carry out examination infrequently [23]. Consistent with our findings, the relationship between incomplete eye closure [2–4, 11] or mechanical ventilation [2, 3, 26] and developing EK is widely documented. The SOFA score was associated with EK development; however, the APACHE II score on admission had little effect. The SOFA score is calculated daily and exclusively takes into account multiple organ dysfunction while the APACHE II score is calculated only once within 24 hours of admission and measures physiological derangement and co-morbidities. Therefore, it may be that organ dysfunction is more important in the development of EK than admission physiological derangement. Due to severe collinearity between sedation and mechanical ventilation, it was statistically impossible to calculate their individual effects independent of each other. Other factors such as P_mean_ and chemosis had no statistically significant effect on the development of EK. This may be because they can be considered as surrogate markers for incomplete eye closure and independently, with complete eye closure, they have little effect on the development of EK. Studies that report an association between chemosis and EK do so only in patients with concurrent eyelid malposition [3, 4].

Many different preventative techniques have been investigated in eye care [15–19], principally moisture chamber techniques and lubrication. However, no one method is shown to be superior [17–19]. Our adopted lubrication method (Lacri-Lube) was popular with the nursing staff due to familiarity and its effectiveness in treating EK in the first phase of the study. The frequency and intensity of intervention for different at-risk groups in the protocol is at the same level as was used for treating EK in those groups during the first phase of the study, thus selecting a very low threshold for preventing EK. Taping the eyelid is an effective method of preventing EK in incomplete eye closure [27]; however, as others [23] and we have found, it can increase the relative’s distress so in our protocol it is restricted to night-time as an option. We have kept the assessment simple and restricted it to the main risk factor identified in the first phase of the study i.e. incomplete eye closure and mechanical ventilation or sedation. A myriad of complex assessment methods have been suggested [22, 28, 29] but given adherence to a protocol is already a major issue [14], a complex assessment is likely to exacerbate the situation.

The strengths of this study are determining the rate of EK and its risk factors in a large sample size with patients having a wide range of severity of illness, daily ophthalmic assessment of all patients during their stay in the ICU using a portable slit lamp, development of a protocol and conducting further study to see the effect of protocolised eye care on developing EK. All of the eye assessments were carried out by one investigator who was fully trained in using a portable slit lamp and recognising eye signs, thus removing interobserver variability. We demonstrated that when a protocol is developed with the involvement of the nursing staff and incorporated into the patient electronic record, excellent adherence and prevention of EK can be achieved. However, the Hawthorne effect of awareness of the study being conducted by the health workers is likely to improve eye care in the first phase and the adherence to the protocol in the second phase of the study.

We did not investigate the long-term visual benefits of preventing EK in the ICU in this study. The study excluded paediatric patients and patients with specialist needs such as those with burns. We did not collect data on the indication for ICU admission or on factors such as use of neuromuscular blocking drugs, fluid balance or renal replacement therapy, so cannot comment on any potential influence of these factors in the development of EK. Although a power calculation was not performed to ascertain the sample size, the results demonstrate that type I or type II error is unlikely to have occurred.

## Conclusions

We conclude that EK is common, but preventable, in critically ill patients. Mechanical ventilation and incomplete eye closure are the major risk factors for development of EK. A simple eye care protocol substantially reduces the incidence of EK, which can be easily achieved in clinical practice when a multidisciplinary focused approach is taken.

## Abbreviations

AOR: Adjusted odds ratio
APACHE II: Acute Physiology and Chronic Health Evaluation II
ED: Epithelial Defect
EK: Exposure keratopathy
GCS: Galsgow Coma Scale
ICU: Intensive Care Unit
MC&S: Microbiology, Culture and Sensitivity
MV: Mechanical Ventilation
OR: Odds ratio
PEE: Punctate Epithelial Erosion
P_mean_: Mean ventilation pressure
RASS: Richmond Agitation-Sedation Scale
SOFA: Sequential Organ Failure Assessment
